# Haplotype-resolved chromosomal-level assembly of wasabi (*Eutrema japonicum*) genome

**DOI:** 10.1038/s41597-023-02356-z

**Published:** 2023-07-11

**Authors:** Hiroyuki Tanaka, Tatsuki Hori, Shohei Yamamoto, Atsushi Toyoda, Kentaro Yano, Kyoko Yamane, Takehiko Itoh

**Affiliations:** 1 School of Life Science and Technology, Tokyo Institute of Technology, Meguro-ku, Tokyo, 152-8550 Japan; 2 Gifu University, Faculty of Applied Biological Sciences, 1-1 Yanagido, Gifu City, Gifu, 501-1193 Japan; 3 Comparative Genomics Laboratory, National Institute of Genetics, Mishima, Shizuoka, 411-8540 Japan; 4 Department of Biological Sciences, Tokyo Metropolitan University, Tokyo, 192-0397 Japan

**Keywords:** Comparative genomics, Polyploidy in plants

## Abstract

In Japan, wasabi (*Eutrema japonicum*) is an important traditional condiment, and is recognized as an endemic species. In the present study, we generated a chromosome-level and haplotype-resolved reference genome for *E. japonicum* using PacBio CLR (continuous long reads), Illumina, and Hi-C sequencing data. The genome consists of 28 chromosomes that contain 1,512.1 Mb of sequence data, with a scaffold N50 length of 55.67 Mb. We also reported the subgenome and haplotype assignment of the 28 chromosomes by read-mapping and phylogenic analysis. Three validation methods (Benchmarking Universal Single-Copy Orthologs, Merqury, and Inspector) indicated that our obtained genome sequences were a high-quality and high-completeness genome assembly. Comparison of genome assemblies from previously published genomes showed that our obtained genome was of higher quality. Therefore, our genome will serve as a valuable genetic resource for both chemical ecology and evolution research of the genera *Eutrema* and Brassicaceae, as well as for wasabi breeding.

## Background & Summary

*Eutrema japonicum* (Miq.) Koidz, wasabi, is endemic to Japan^1^. It serves as a traditional and crucial condiment in the consumption of ‘sushi’ or ‘soba’ in Japan. The major wasabi variety “Mazuma” boasts both a giant rhizome and a potent pungency, and is currently the most valuable cultivar. Wasabi is a member of the Brassicaceae family, which includes numerous scientifically and commercially significant species^1^. Currently, the National Center for Biotechnology Information (NCBI) genome database has sequenced eighty-seven species of the Brassicaceae family. Within the genus *Eutrema*, whole-genome sequences have been released for the wild species *Eutrema heterophyllum* (W. W. Smith) H. Hara, and *Eutrema yunnanense* Franch^2^. However, in Japan, only the chloroplast genomes of *Eutrema* species, including wasabi, have been sequenced^3^. The Brassicaceae family is well known for its high frequency of hybridization^4^ and polyploidy, or whole-genome duplication (WGD) events, with over 43% of species exhibiting such characteristics^5^. These features may have played a crucial role in the diversification and adaptive survival of the species, particularly during the Quaternary period^6^. The chromosome number of wasabi was 28^7^, and it is a tetraploid, similar to that of *E. yunnanense* (2n = 4x = 28)^7^. While wasabi polyploidy has been posited, it is yet to be confirmed in Japan, and its genome constitution has not been reported (i.e. allo- or auto-polyploid).

Additionally, the phylogenetic relationships between *E. yunnanense*, a close relative to wasabi, and *Eutrema* species in Japan are yet to be investigated using nuclear DNA polymorphisms.

Recent advancements in long-read sequencing have enabled the acquisition of uninterrupted genomic sequence data from highly heterozygous plant species^8^. In this study, we provide the first report of whole-genome sequences of wasabi, and investigate its chromosome structure using chromosome-level de novo genome sequencing. We also directly compared the genomes of other *Eutrema* species. We anticipate that our data will provide the genetic basis for understanding the variable pungent components and defense systems, as well as the evolutionary history of wasabi as an endemic plant in Japan. Our data will serve as a valuable genetic resource for both chemical ecology and evolutionary research of the genus *Eutrema*, and family Brassicaceae, as well as for wasabi breeding.

## Methods

### Sample collection and sequencing

**Fig. 1:**
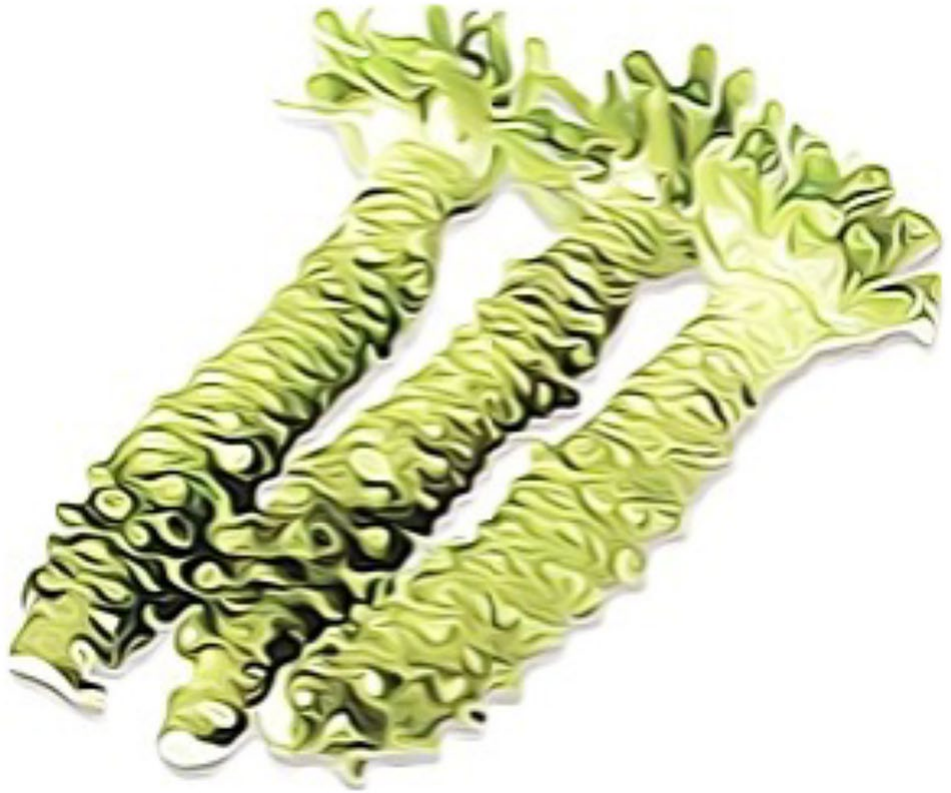
The variety ‘Mazuma’ No.3 of Wasabi, *Eutrema japonicum*.

The variety “Mazuma” No. 3 (wasabi, Fig. 1) from MIYOSHI AGRI-TECH CO., LTD. was selected for the whole genome sequencing of the wasabi. Individual used for genome sequencing is stored in the Laboratory of Plant Genetics and Breeding, Faculty of Applied Biological Sciences, Gifu University. Whole-genome shotgun sequencing was performed using the PacBio and Illumina sequencing platforms. Genomic DNA from *Eutrema japonicum* was isolated using a NucleoBond® HMW DNA kit (Macherey-Nagel, Germany) and sheared into fragment sizes ranging from 30 kb to 100 kb with a g-tube device (Covaris Inc., MA, USA). A Continuous Long Read (CLR) SMRTbell library was prepared using a SMRTbell Express Template Prep Kit 2.0 (Pacific Bioscience, CA, USA) according to the manufacturer’s instructions. The CLR library was size-selected using the BluePippin system (Saga Science, MA, USA) with a lower cutoff of 30 kb. One SMRT Cell 8 M was sequenced on the PacBio Sequel II system with Binding Kit 2.0 and Sequencing Kit 2.0 (20 h collection times). As a result of PacBio sequencing, 175.73 Gb of CLR reads were obtained (Table 1). In addition, genomic DNA was fragmented to an average size of 600 bp using a M220 Focused-ultrasonicator (Covaris Inc., MA, USA). A paired-end library with insert sizes from to 550–650 bp was constructed using a TruSeq DNA PCR-Free Library Prep kit (Illumina, CA, USA) and was size-selected on an agarose gel with a Zymoclean Large Fragment DNA Recovery Kit (Zymo Research, CA. USA). The final library was sequenced using a 2 × 250 bp paired-end protocol for the HiSeq. 2500 system (Illumina, San Diego, CA, USA). As a result of Illumina sequencing, we obtained 73.47 Gb of Illumina paired-end reads (Table 1). The Arima Hi-C library was constructed using an Arima-HiC + Kit (Arima Genomics, CA, USA) according to the manufacturer’s instructions for Plant Tissues (A160135 v01) and library preparation was done using the KAPA Hyper Prep Kit (A160139 v00). In brief, 2.0 grams of the frozen leaves were crosslinked with 37% formaldehyde solution under a vacuum for 25 min and stopped in 125 mM glycine. Crosslinked leaves were ground into powder in liquid nitrogen, and 1.8 g of the powder was suspended in 6.0 mL nuclei isolation buffer. Nuclear extraction was performed using a CelLytic PN Isolation/Extraction Kit (Sigma-Aldrich, MO, USA) according to the manufacturer’s recommendations for cell lysis and semi-pure preparation of nuclei. The cross-linked DNA was digested with two restriction enzymes (^GATC and G^ANTC). After incorporating biotinylated nucleotides into the digested DNA ends, both ends were ligated to the spatially proximal ends. To reverse formaldehyde cross-linking, the crosslinked DNA was incubated as follows: 55 °C for 30 min, 68 °C for 90 min, and 25 °C for 10 min. The ligated DNA was mechanically sheared into average sizes of 400–500 bp using the M220 focused ultrasonicator, and the ligation junctions were enriched with streptavidin magnetic beads. The sequencing library was prepared from enriched DNA fragments with a KAPA Hyper Prep Kit (Roche Molecular Systems, CA, USA) and amplified for six PCR cycles. The concentration and quality of the libraries were evaluated using a Qubit 4 Fluorometer (Thermo Fisher Scientific, MA, USA), 2100 Bioanalyzer system (Agilent Technologies, CA, USA), and 7900HT Fast Real-Time PCR System (Thermo Fisher Scientific, MA, USA). The final Hi-C libraries were run on the Illumina NovaSeq. 6000 system (Illumina, San Diego, CA, USA) with 2 × 150 bp read length, and 160.84 Gb of Arima Hi-C reads were generated (Table 1). The Omni-C library was prepared using the Dovetail Omni-C Kit (Dovetail Genomics, Scotts Valley, CA, USA) according to the manufacturer’s protocol. Approximately 300 mg of leaf tissue was ground in liquid nitrogen with a mortar and pestle. The pulverized material was processed into a proximity ligation library using the Omni-C Proximity Ligation Assay Non-Mammalian Sample Protocol v1.2 of the Omni-C Kit (Dovetail Genomics, Scotts Valley, CA, USA). The final library was sequenced on a NovaSeq 6000 instrument with 2 × 100 bp read length, and 82.53 Gb of Omni-C reads were generated (Table 1).

**Table 1:** Sequencing data used for the *E. japonicum* genome assembly.

Library types	Insert size (bp)	Reads number	Raw data (Gb)	Average length (bp)	N50 length (bp)
Illumina paired-end	550	293,861,760	73.47	250	—
Pacbio CLR	≥30,000	6,699,890	175.73	26,229.36	40,362
Hi-C (Arima)	—	1,072,258,958	160.84	150	—
Hi-C (Omni-C)	—	825,255,480	82.53	100	—

### Genome size and heterozygosity estimation

**Fig. 2:**
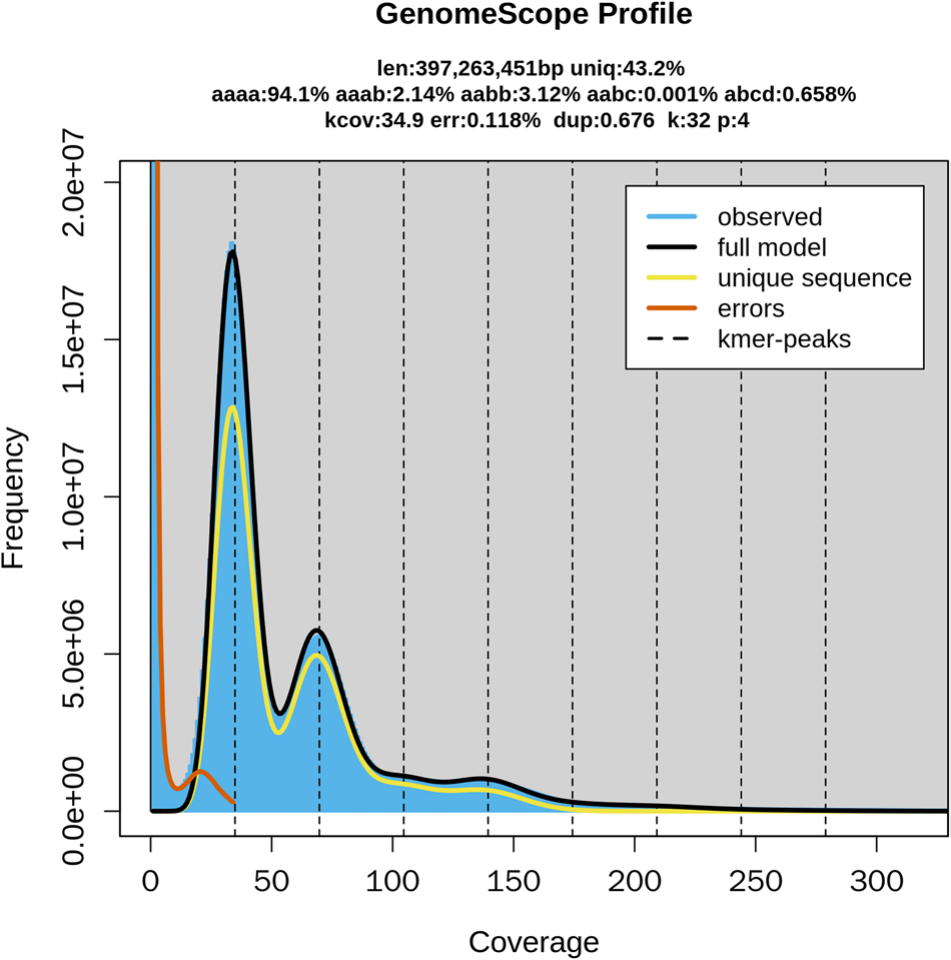
K-mer analysis of *E. japonicum* genome.

The genome size of *E. japonicum* was estimated from Illumina sequencing data using the k-mer-based method^9^. Illumina sequenced reads were filtered using platanus_trim v1.0.7 (http://platanus.bio.titech.ac.jp/pltanus_trim) with default parameters. To remove highly copied reads derived from the chloroplast genome, filtered reads were mapped to the *E. japonicum* chloroplast genome^10^, and unmapped reads were extracted. Using the unmapped reads, Jellyfish v2.2.10^11^ was first applied to extract and count canonical k-mers at k = 32. Subsequently, GenomeScope 2.0^9^ was used to estimate haploid genome size and heterozygosity from k-mer count data with parameters of “-k 32 -p 4” (Fig. 2). As a result, we estimated a haploid genome size of 397.3 Mb with a heterozygosity of 5.9% (aaab: 2.14%, aabb: 3.12%, aabc: 0.01%, abcd: 0.658%, respectively); therefore, the homozygosity rate was estimated to be 94.1% (Fig. 2).

### Chloroplast genome assembly

Illumina sequenced reads were filtered using platanus_trim v1.0.7 (http://platanus.bio.titech.ac.jp/pltanus_trim) with the following parameters “-q 0.” The trimmed reads were assembled using NOVOPlasty v4.3.1^12^ with the following parameters: “Type = chloro, K-mer = 36, Read Length = 250, Insert size = 600, Platform = illumina, Single/Paired = PE, Insert size auto = yes”. The chloroplast genome of *E. japonicum* was used as the reference^10^, and *psbA* chloroplast gene sequences were used as seeds to assemble the plastome. As a result, a chloroplast genome of 153,794 bp was obtained^13,14^.

### Mitochondrial genome assembly

Firstly, we performed a de novo assembly of the trimmed reads using NOVOPlasty v 4.3.1^12^ with the following parameters: “Type = mito_plant, K-mer = 36, Read Length = 250, Insert size = 600, Platform = illumina, Single/Paired = PE, Insert size auto = yes”. Then, the *cox1*^15^ and *nad6*^15^ genes were used for the seed sequence in an independent assay. Preliminary draft mitochondrial contigs were obtained. We used mitochondrial contigs as bait files to extract PacBio reads from the sequenced mitochondrial genome. Then, the extracted PacBio reads were assembled using Flye v2.9-b1768^16^ and polished using Racon v1.4.20^17^. PacBio-based assemblies were used as the new bait file to extract Illumina reads from the sequenced mitochondrial genome. Finally, using both the extracted Illumina and PacBio reads, we applied Unicycler v0.4.8^18^ to perform a hybrid assembly. We obtained two independent contigs, totaling 309,141 bp. The length of the longer contig was 201,312 bp^14,19^, while that of the shorter contig was 107,829 bp^14,20^.

### *De novo* genome assembly

PacBio sequenced reads were used for genome assembly by the Canu v2.1.1^21^ with parameters of “genomeSize = 600 m corOutCoverage = 200 “batOptions = -dg 3 -db 3 -dr 1 -ca 500 -cp 50” correctedErrorRate = 0.035 -pacbio-raw”. The draft assembly contigs were polished with two rounds of Arrow (Pacific Biosciences) and three rounds of HapoG v1.3.4^22^ and then the Arrow-identified variants were filtered via Merfin^23^ using Illumina sequenced reads. Organelle contigs were identified by alignment against the already generated mitochondrial and chloroplast genomes using nucmer v4.0.0beta2^24^. After removing organelle contigs, redundant contigs, short contigs (<15,000 bp), and contigs of aberrant GC content (<1% or >99%), we obtained 616 contigs with a total size of 1,529.3 Mb, which was approximately four times the estimated haploid genome size (397.3 Mb). This result suggested that not only the subgenome but also each of the haplotype genomes that make up the tetraploid chromosomes are constructed into contigs in a separate form.

### Chromosome assembly using Hi-C data

**Fig. 3:**
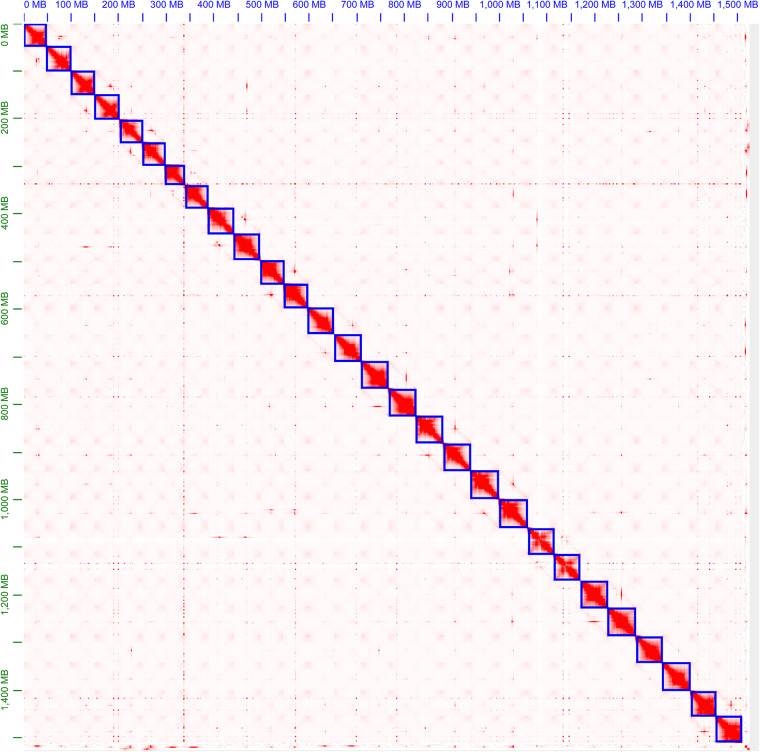
Genome-wide Hi-C heatmap of *E. japonicum*. The blue squares represent chromosomes.

To obtain a haplotype-resolved chromosome assembly of *E. japonicum*, we performed Hi-C scaffolding using two different Hi-C datasets (Arima Hi-C and Omni-C). The Arima Hi-C reads were mapped to the cleaned contigs and processed to generate Hi-C contacts by Juicer v1.6^25^ with the following parameter settings: “-s Arima.” Bridge sequences were trimmed from the Omni-C reads using cutadapt v4.1^26^. The cleaned Omni-C reads were then processed using the Juicer pipeline with default parameters. Two distinct Hi-C contacts files (merged_nodups.txt) were combined and used for scaffolding by the 3D-DNA v180922^27^ with the following parameter settings: “--rounds 0 --editor-coarse-resolution 2500000 --editor-coarse-region 12500000 --editor-coarse-stringency 1 --polisher-coarse-resolution 2500000-- polisher-coarse-region 15000000 --polisher-coarse-stringency 1 --splitter-coarse-resolution 2500000 --splitter-coarse-region 15000000 --splitter-coarse-stringency 1,” so as to avoid unnecessary fragmentation. We visualized the Hi-C contact map and performed extensive manual curation using Juicebox v1.11.08^28^ to fix mis-assemblies and mis-scaffoldings. After manual curation, overlapping contig ends (identity ≥99%) of flanking contigs were merged using an in-house script (https://github.com/th2ch-g/Canu-Contig-Overlap-Merge). As a result, Hi-C data helped to anchor 477 contigs of 1,512.1 Mb sequence to 28 chromosomes (Fig. 3). The scaffold N50 size was 55.67 Mb (Table 2).

**Table 2:** Assembly statistics of *E. japonicum* genome.

Genome assembly statistics	Scaffold	Chromosome
Total length	1,525,751,942	1,512,080,002
Number of Contig	167	28
Average length (bp)	9,136,239.2	54,002,857.2
Max length (bp)	60,636,194	60,636,194
N50 length (bp)	55,670,424	55,670,424
Completeness BUSCOs (%)	99.24	99.24
Complete single-copy BUSCOs (%)	0.61	0.61
Complete duplicated BUSCOs (%)	98.63	98.63
Complete duplicated BUSCOs (%)	0.04	0.04
Complete duplicated BUSCOs (%)	0.72	0.72
Merqury completeness	48.78	51.33
Merqury QV	97.89	97.82
Inspecter Mapping rate	91.98	91.99
Inspecter QV	43.95	43.46

### Chromosome, sub-genome and haplotype assignment

**Fig. 4:**
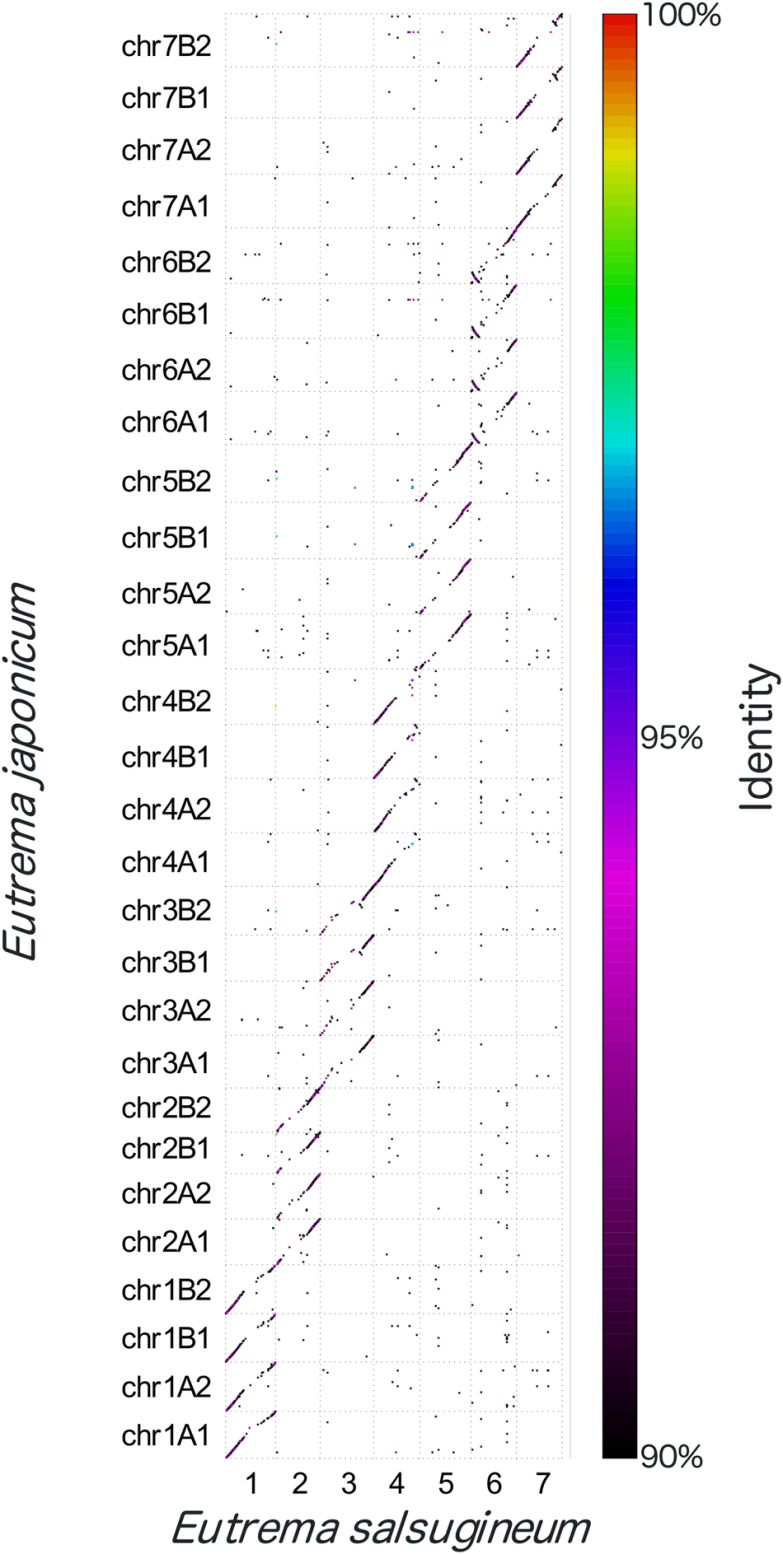
Comparison of sequence similarity with *E. salsugineum*. Dot plot analysis comparing *E. japonicum* homoeologous chromosomes and *E. salsugineum* chromosomes.

**Fig. 5:**
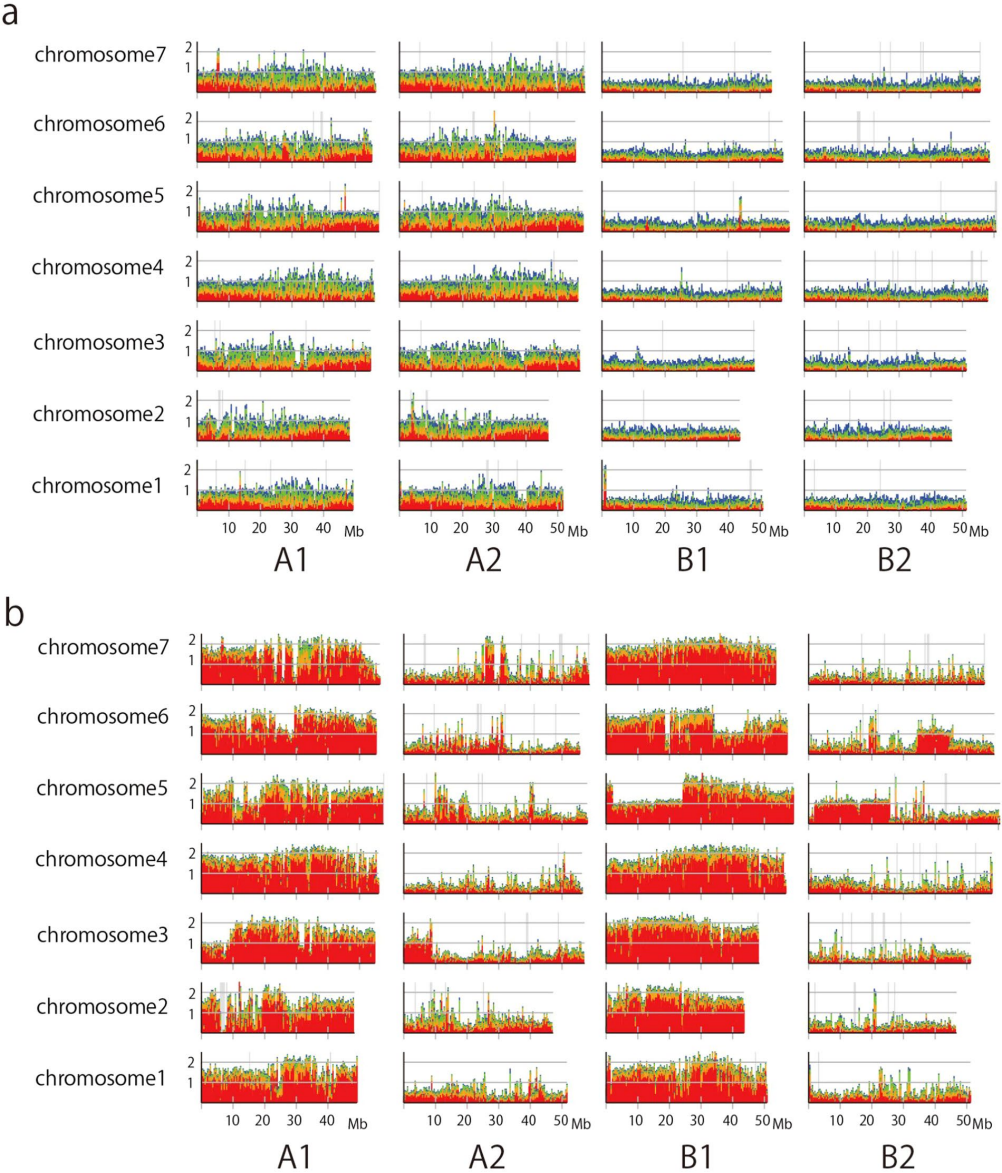
Mapping analysis of other *Eutrema* sequence reads against chromosomes of *E. japonicum*. The normalized sequencing depth of the diploid *E. yunnanense* reads (**a**) and previously published *E. japonicum* reads (**b**) are shown. Coverage corresponding to haploid is set to 1. Colored by identity of mapped read (Red: 99%≥ identity, Orange: ≥97.5% identity, Green: ≥95% identity, Blue: ≥90% identity).

A comparison of sequence similarity with *Eutrema salsugineum* enabled the identification of homoeologous sets of *E. japonicum* chromosomes, with each of the four chromosomes showing clear sequence similarity to each *E. salsugineum* chromosome as a set (Fig. 4). To assign chromosomes within each homoeologous set to the A or B sub-genome, we mapped and visualized the location of sequencing reads from diploid *E. yunnanense*^2,29–31^ and *E. japonicum*^32,33^. Fourteen chromosomes showed a clear overabundance of mapped diploid *E. yunnanense* reads (Fig. 5) and were assigned to subgenome A. The remaining 14 chromosomes were assigned to the B sub-genome. Subsequently, seven chromosomes belonging to the A sub-genome and seven chromosomes belonging to the B sub-genome showed a clear overabundance of mapped *E. japonicum* reads (Fig. 5) and were assigned to haplotype A1 and haplotype B1, respectively. The remaining chromosomes belonging to the A sub-genome and 7 chromosomes belonging to the B sub-genome were assigned to haplotype A2 and haplotype B2, respectively.

### Intra-chromosomal comparative genome analysis

**Fig. 6:**
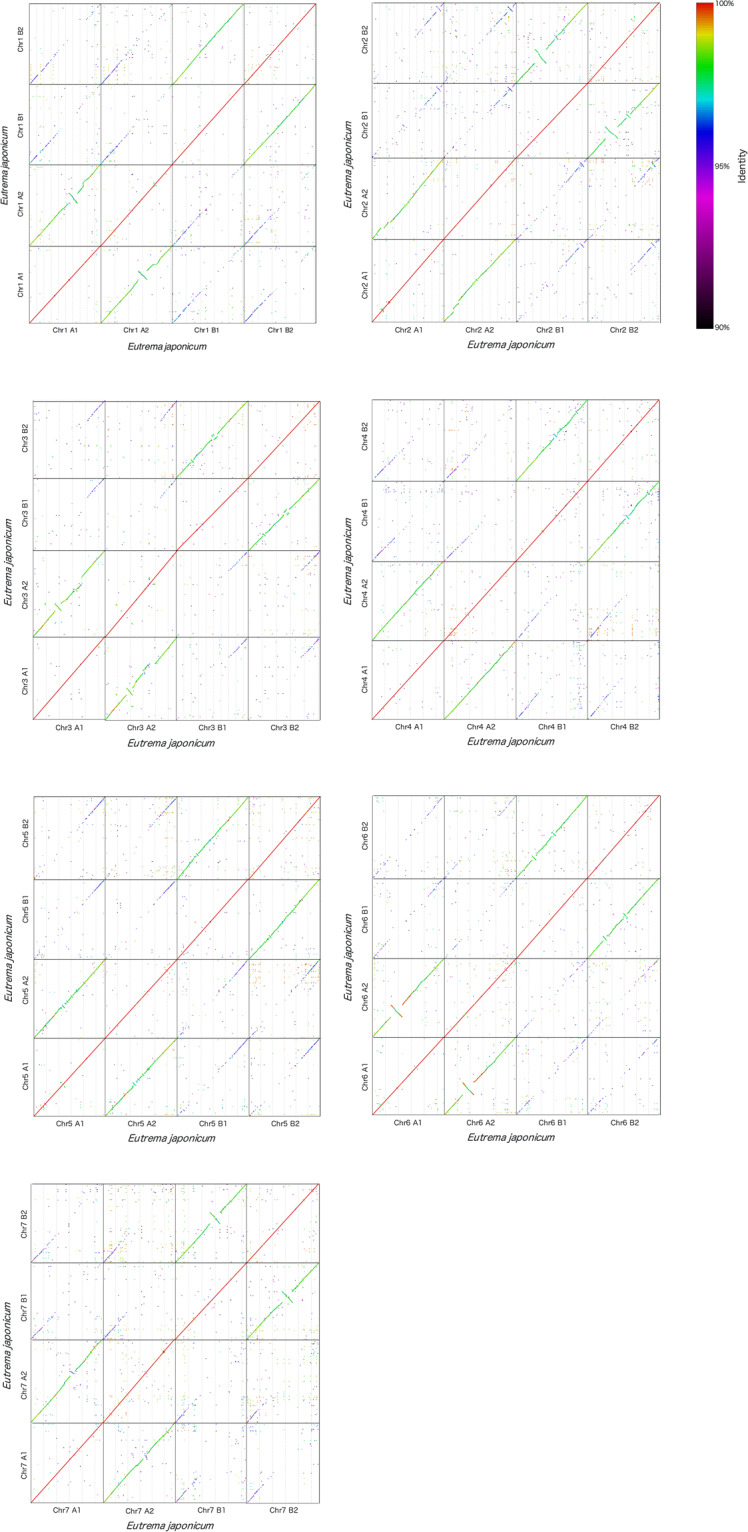
Self Dot-plot analysis of four haplotype-resolved *E. japonicum* chromosomes. X-axis: From left to right: Chromosomes A1, A2, B1, and B2. Y-axis: From bottom to top: Chromosomes A1, A2, B1, and B2.

Within each of the seven homoeologous chromosome groups, the chromosome-level sequences of the four haplotypes were aligned to each other using nucmer v4.0.0beta2^24^ with default parameters (Fig. 6) and visualized using a mummerplot and gnuplot. High collinearity was detected between all homoeologous chromosomes, but several large genomic inversions (>5 Mbp) were detected between chromosomes within the subgenome, for example, chr1A1 vs. chr1A2 and chr1B1 vs. chr1B2 (Fig. 6). To validate the predicted inversion, we selected six candidate inversion loci and performed PCR validation, which resulted in missed assemblies that were not observed. A high-confidence alignment subset (filtered with delta-filter -q -r) was used to calculate the average genome sequence similarity. The average genome sequence similarity between the chromosomes within the subgenome is 97.6–98.0%, while inter-subgenome homoeologous chromosomes are 95.7–96.3%.

### Genome sequence feature (repetitive sequence analysis)

**Fig. 7:**
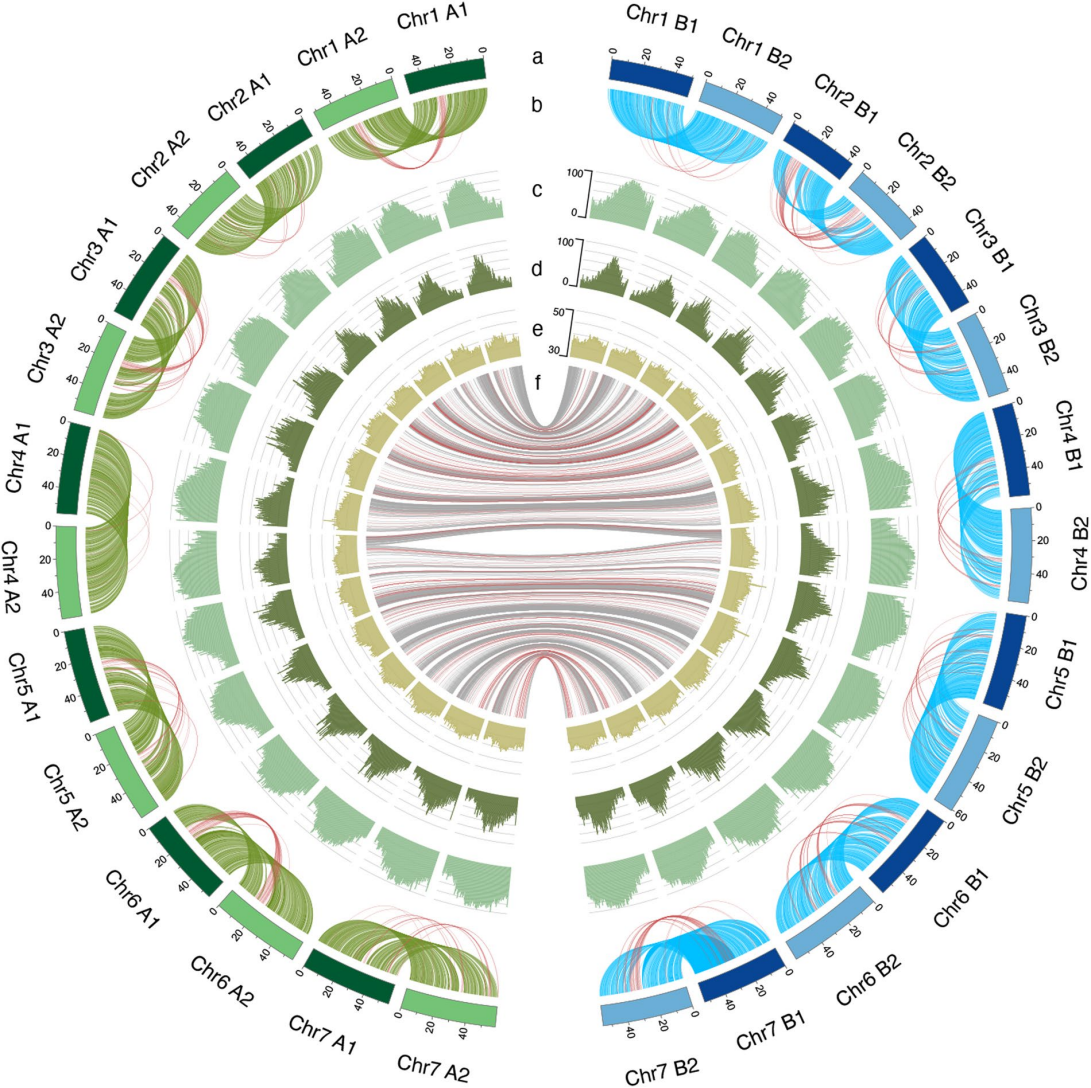
Characterization of the *E. japonicum* genome. From the outer to the inner layers: chromosomes with scales (**a**), links between intra-subgenomes syntenic blocks (**b**), repeat element abundance (**c**), LTR element abundance (**d**), GC rate (**e**), and links between inter-subgenome syntenic blocks (**f**) are shown. The links between intra-subgenome syntenic blocks (**b**) are indicated by green lines for subgenome A and by cyan lines for subgenome B, and the links between inter-subgenome syntenic blocks (**f**) are indicated by gray lines. The links between inverted syntenic blocks are indicated by red lines (**b** and **f**).

To identify the repetitive elements in the *E. japonicum* chromosome assembly, RepeatModeler v2.0^34^ was used to construct a repetitive library, followed by RepeatMasker v4.0.9 (http://www.repeatmasker.org). For RepeatModeler, in addition to the default parameters, the parameters, the following parameter “-LTRStruct” was used. For RepeatMasker, the additional parameters were “a” and “gff.” Repeats accounted for 66.9% of the genome, with LTR-retrotransposons representing the most abundant repeat class (41.0%). Other repeats, including LINEs, SINEs, and DNA elements, made up only minor genome proportions (Table 3). The 28 chromosomes were all characterized by a high density of repetitive sequences and transposable elements in the pericentromeric and centromeric regions (Fig. 7).

**Table 3:** Repeat annotation in the *E. japonicum* genome.

Type	Length (bp)	% in genome
SINEs	3,542,957	0.23
LINEs	43,684,203	2.89
LTR elements	620,050,949	41.01
DNA elements	192,257,396	12.71
Unclassified	113,781,013	7.52

### Comparative genomic analysis

**Fig. 8:**
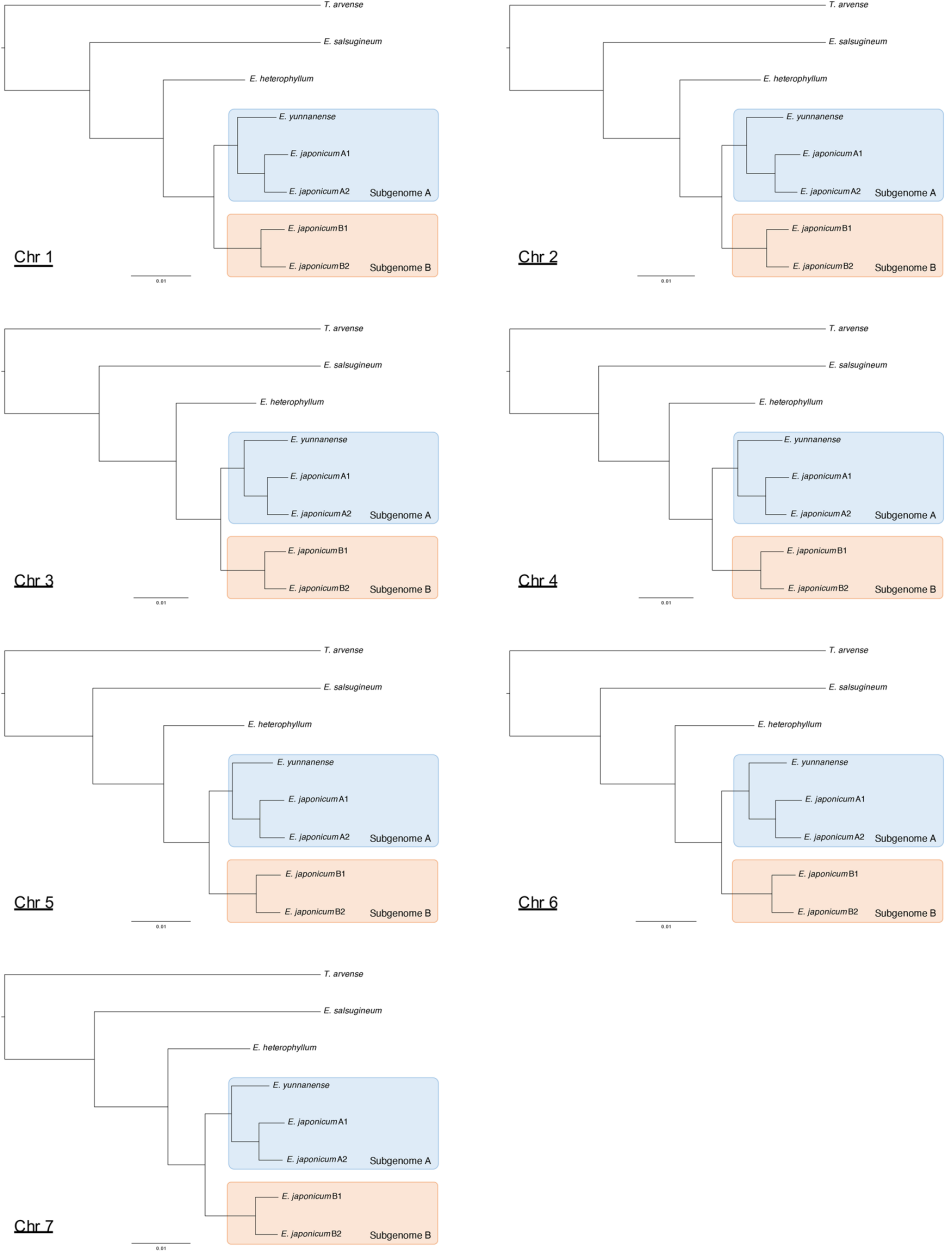
The phylogenetic analysis of *E. japonicum* and closely related species. As ortholog groups, 515, 415, 333, 439, 534, 448, and 443 groups were used for chromosomes 1, 2, 3, 4, 5, 6, and 7, respectively. *T. arvense* was used as an outgroup.

To investigate the phylogenetic relationships of the four haplotype-resolved sets of *E. japonicum* chromosomes with related species, comparative genomic analysis was conducted based on orthologous gene information. For comparison, we selected the following plant species as targets. The genome sequences of *Thlaspi arvense*^35^, *E. salsugineum*^36^, *E. heterophyllum*^37^, and *E. yunnanense*^38^ were downloaded from NCBI GenBank. Then, Benchmarking Universal Single-Copy Orthologs (BUSCO)^39^ analysis with the brassicales_odb10 database against each genome sequence was conducted. Based on the BUSCO gene id information predicted by this analysis, 2,524 ortholog groups were extracted with a one-to-one gene relationship across all eight genomes (*T. arvense, E. salsugineum, E. heterophyllum, E. yunnanense, E. japonicum* haplotype-A1, *E. japonicum* haplotype-A2, *E. japonicum* haplotype-B1, and *E. japonicum* haplotype-B2). For each ortholog group, multiple alignments were performed with MAFFT v7.407^40,41^, and sites containing gaps (“−”) or ambiguous characters (“X”) were excluded. All alignments were concatenated and used for phylogenetic analysis. A phylogenetic tree was constructed using RAxML v8.2.12^42^. We applied the JTT substitution matrix using a gamma model of rate heterogeneity (-m PROTGAMMAJTT). Phylogenetic analysis performed with each chromosome dataset showed a similar topology (Fig. 8). Four *E. japonicum* haplotypes were placed into two separate clades (subgenome A and subgenome B), and diploid *E. yunnanense* was included in the subgenome A clade (Fig. 8).

## Data Records

The genomic sequencing data (Illumina, PacBio, Hi-C) are available in the NCBI SRA database under BioProject ID PRJDB15095. The accession number of Illumina sequencing data is DRR438370^43^. The accession number of PacBio sequencing data is DRR433109^44^. The accession number of Hi-C sequencing data are DRR439365^45^ and DRR439366^46^. The final chromosome assemblies are available in the NCBI GenBank database under BioProject ID PRJDB15185 and PRJDB15186. The accession number of chromosome assembly haplotype-1 (principal haplotype; subgenome A1 and subgenome B1) is BSQW01000001-BSQW01000014^47^. The accession number of chromosome assembly haplotype-2 (alternate haplotype; subgenome A2 and subgenome B2) is BSQX01000001- BSQX01000014^48^. The organelle genome assemblies are available in the NCBI GenBank database and FigShare database^14^. The accession number of chloroplast genome is LC770997.1^13^. The accession number of mitochondrial genomes are LC770998.1^19^ and LC770999.1^20^. Other data, such as structure annotation of BUSCO genes, predicted CDS and protein sequences of BUSCO genes, and annotation of TEs, are available at FigShare database^14^.

## Technical Validation

### DNA quality

Agarose gel electrophoresis and Pippin Pulse (Saga Science, MA, USA) were used to confirm the absence of total RNA and the fragment size of the purified DNA molecules. The concentration was measured using the Qubit 4 Fluorometer (Thermo Fisher Scientific, MA, USA). The main bands of genomic DNA fragments were over 40 kb, and the Nanodrop ND-1000 DNA spectrophotometer (LabTech, Corinth, MS, USA) ratio (260/280) was 1.82.

### Assembly evaluation

The quality and completeness of chromosome assembly were evaluated using three independent approaches. First, the QV value and completeness were estimated using Merqury v1.3^49^ by comparing k-mers in the assembly to those found in the Illumina sequence reads. The results revealed that the QV value for chromosome assembly was 51.33, and the completeness value was 97.82%. Secondly, the completeness of the chromosome assembly was also assessed using BUSCO v5.4.3^39^ with 4,596 single-copy orthologs from the brassica_odb10 database. BUSCO analysis identified 99.24% (4, 561) complete BUSCOs (0.61% single-copy and 98.63% duplicated BUSCOs) and 0.04% (2) fragmented BUSCOs in the genome of *E. japonicum*. Thirdly, we further evaluated the assembly quality using Inspector^50^ by aligning PacBio sequence reads to the assembled contigs to generate read-to-contig alignment and performed downstream assembly evaluation. The read-to-contig mapping rate and QV value were 91.98% and 43.95, respectively. All these indicators suggest a high-quality and high-completeness genome assembly for further genetic research of *E. japonicum*.

Comparison of genome assembly and gene models with the previous study

A comparison was performed between the *E. japonicum* genome and the previously published genom^32^. The scaffold N50 size of our *E. japonicum* genome was 55.67 Mb, which was significantly longer than the previously published assembly (N50 = 356 kb). Regarding its quality, our assembly showed that the BUSCO evaluation achieved 99.24% completeness, whereas the previous result was extremely low at 67.35%. Therefore, these results indicate that our *E. japonicum* genome was of higher quality than the previously published genome.

## Data Availability

The code is available at Github (https://github.com/th2ch-g/Canu-Contig-Overlap-Merge) for merging overlapped contig ends. Other software and pipelines were executed according to the manual and protocols of the published bioinformatic tools. The version and code/parameters of software have been described in Methods.
